# Supplementary material for: The Threshold Effect of Swine Epidemics on the Pig Supply in China
Source: Animals (Basel). 2022 Sep 28;12(19):2595. doi: 10.3390/ani12192595 (PMC9558980; doi:10.3390/ani12192595)
Supplement: Supplementary file 1 [file animals-12-02595-s001.zip › animals-1865586-supplementary.pdf]

Supplementary data

Table S1. Data used in this article.

Date	ISEW	Pig stock	Pig slaughter	Piglet price	Chicken price	Feeding cost	Profit	Pig price	Sow price	Corn price
2012.01	0.38	46467.00	5755.98	29.53	15.88	755.25	276.43	15.07	1610.62	2.15
2012.02	0.39	45846.00	5839.12	31.95	15.89	799.25	235.38	14.89	1634.74	2.17
2012.03	0.39	46167.00	5922.25	33.09	15.91	760.61	182.72	14.47	1609.50	2.17
2012.04	0.30	46305.50	6005.39	30.26	16.19	773.87	-20.81	13.73	1614.23	2.19
2012.05	0.31	46120.30	5920.99	24.67	16.12	771.22	-221.42	13.38	1630.04	2.20
2012.06	0.28	46212.50	5836.59	24.38	16.02	775.22	-118.18	12.59	1641.07	2.21
2012.07	0.26	46212.50	5752.20	26.20	15.83	792.31	-80.41	11.89	1597.09	2.20
2012.08	0.24	46628.40	5751.60	23.84	15.85	819.29	-18.71	11.28	1451.72	2.20
2012.09	0.27	47234.60	5751.00	24.69	15.87	824.05	78.37	11.72	1442.57	2.21
2012.10	0.25	47281.80	5750.40	23.85	15.79	819.50	92.94	12.09	1490.33	2.18
2012.11	0.25	46903.60	5800.47	22.46	15.62	793.58	101.89	12.28	1484.90	2.17
2012.12	0.27	46340.70	5850.54	23.54	15.52	811.40	208.72	13.16	1540.16	2.16
2013.01	0.29	44857.80	5900.62	26.39	15.49	821.91	318.99	14.47	1603.69	2.19
2013.02	0.33	44005.50	5925.42	25.42	15.53	820.46	268.19	13.15	1623.96	2.18
2013.03	0.31	44401.60	5950.23	20.77	15.69	808.76	4.62	12.37	1508.91	2.20
2013.04	0.29	44712.40	5975.04	20.15	15.83	801.05	-125.69	11.57	1431.36	2.18
2013.05	0.26	44801.80	5967.57	20.55	15.96	796.17	-118.93	11.74	1425.50	2.15
2013.06	0.25	45249.80	5960.10	21.95	16.12	803.19	49.17	12.29	1425.71	2.10
2013.07	0.25	45792.80	5952.63	23.16	16.05	813.11	133.46	12.16	1446.38	2.09
2013.08	0.25	46205.00	5970.08	24.09	15.82	813.28	208.61	12.62	1459.83	2.08
2013.09	0.25	46528.40	5987.52	23.85	15.57	819.91	321.34	12.54	1442.45	2.08
2013.10	0.26	46807.60	6004.97	21.30	15.44	811.68	96.79	12.55	1401.81	2.11
2013.11	0.28	46901.20	6035.10	20.15	15.39	794.19	198.41	12.79	1411.40	2.13
2013.12	0.30	45775.60	6065.23	20.21	15.31	793.61	230.20	12.60	1451.15	2.12
2014.01	0.31	43853.00	6095.36	16.72	15.22	777.37	29.60	11.03	1302.95	2.10
2014.02	0.37	43458.00	6126.99	15.10	15.04	765.91	-129.03	10.60	1264.11	2.09
2014.03	0.33	43455.00	6158.61	15.11	14.76	765.65	-219.63	10.31	1227.08	2.08
2014.04	0.28	42890.10	6190.24	15.83	14.17	776.34	-250.65	9.68	1150.52	2.06
2014.05	0.26	42847.20	6172.16	19.91	14.16	814.82	-30.82	11.53	1242.56	2.06
2014.06	0.26	42932.90	6154.09	20.71	13.79	830.04	-7.21	10.85	1257.14	2.09
2014.07	0.27	43061.70	6136.01	20.17	13.84	837.47	62.74	10.98	1214.38	2.13
2014.08	0.27	43320.10	6116.10	23.43	14.02	882.70	165.78	11.36	1248.53	2.17
2014.09	0.28	43666.60	6096.19	24.02	14.40	851.58	178.66	11.34	1292.96	2.21
2014.10	0.28	43579.30	6076.28	21.75	14.78	777.20	129.74	11.43	1327.16	2.18
2014.11	0.29	43187.10	6067.22	20.93	14.88	765.48	141.33	11.37	1375.22	2.15
2014.12	0.30	42193.80	6058.16	17.74	14.89	759.03	84.15	10.83	1390.83	2.11
2015.01	0.31	40590.40	6049.10	17.60	15.01	758.81	-23.56	10.84	1384.53	2.10
2015.02	0.31	39007.40	5960.43	17.35	15.07	763.52	-128.52	10.53	1355.76	2.08
2015.03	0.30	38734.30	5871.75	19.15	15.17	800.28	-207.39	10.79	1358.49	2.09
2015.04	0.29	38726.60	5783.07	20.13	15.24	800.62	8.81	11.62	1392.99	2.09
2015.05	0.26	38649.10	5778.28	22.82	15.04	799.00	125.12	12.47	1484.51	2.08
2015.06	0.26	38494.50	5773.49	25.31	14.99	796.26	258.53	12.62	1503.30	2.06
2015.07	0.25	38571.50	5768.71	29.57	14.97	801.65	471.88	14.20	1620.13	2.02
2015.08	0.26	38764.40	5816.71	33.27	15.08	790.45	596.97	13.98	1683.70	1.98
2015.09	0.32	38997.00	5864.71	30.16	15.12	720.25	581.96	13.62	1672.07	1.92
2015.10	0.25	39114.00	5912.71	25.65	15.28	632.70	512.44	13.54	1631.20	1.85
2015.11	0.30	38840.20	5807.90	25.05	15.47	658.35	390.69	13.07	1626.41	1.80
2015.12	0.23	38412.90	5703.08	25.17	15.65	655.96	354.58	13.15	1664.77	1.80
2016.01	0.22	37375.80	5598.26	26.88	15.69	632.70	508.50	14.42	1701.58	1.78
2016.02	0.26	36703.00	5614.96	30.05	15.65	629.28	547.35	15.22	1801.82	1.76
2016.03	0.30	37033.30	5631.65	37.52	15.48	599.36	791.10	16.75	1984.42	1.71

2016.04	0.29	37255.50	5648.35	44.37	15.71	586.19	886.38	17.44	2163.34	1.65
2016.05	0.24	37404.60	5691.41	47.31	15.82	604.49	889.53	18.27	2339.72	1.63
2016.06	0.24	37516.30	5734.47	46.27	16.14	632.70	856.80	17.11	2310.81	1.66
2016.07	0.26	37478.80	5777.54	43.29	16.35	634.75	544.62	15.10	2118.89	1.69
2016.08	0.23	37553.80	5786.62	42.51	16.58	604.49	450.80	13.96	2000.90	1.65
2016.09	0.19	37591.30	5795.70	41.92	15.72	588.92	362.36	13.52	1967.81	1.63
2016.10	0.18	37591.30	5804.78	36.71	15.29	564.30	143.08	12.70	1796.77	1.59
2016.11	0.20	37365.80	5747.83	35.34	15.19	569.43	263.91	13.04	1803.22	1.60
2016.12	0.24	36618.40	5690.88	34.98	14.99	556.78	342.82	13.28	1806.19	1.60
2017.01	0.15	35556.50	5633.92	38.33	14.83	541.22	451.77	14.29	1866.61	1.56
2017.02	0.18	35485.40	5636.51	36.92	17.93	539.51	433.55	13.74	1903.99	1.54
2017.03	0.17	35840.30	5639.09	35.75	17.67	543.78	269.08	13.67	1835.50	1.52
2017.04	0.16	35983.60	5641.68	33.83	18.23	549.77	299.75	13.12	1812.28	1.52
2017.05	0.19	35443.90	5701.09	30.18	18.60	555.32	106.42	12.19	1757.46	1.52
2017.06	0.20	35373.00	5760.50	29.80	18.53	555.75	78.55	11.28	1716.96	1.53
2017.07	0.22	35125.40	5819.91	30.02	18.26	575.42	71.12	11.14	1667.87	1.54
2017.08	0.21	34949.70	5836.49	30.52	17.76	560.03	158.13	10.82	1584.45	1.54
2017.09	0.21	34879.80	5853.07	30.84	18.51	562.93	242.19	10.65	1617.28	1.56
2017.10	0.20	34810.10	5869.65	29.44	18.59	552.33	232.74	10.85	1602.39	1.57
2017.11	0.20	34775.30	5875.79	29.90	18.71	550.62	235.59	10.79	1610.89	1.57
2017.12	0.26	34045.00	5881.93	30.07	19.08	571.82	271.81	11.24	1611.33	1.57
2018.01	0.17	35521.00	5888.07	30.12	19.31	627.57	235.03	11.65	1638.61	1.60
2018.02	0.16	35201.30	5816.98	28.43	19.64	636.12	108.25	10.22	1668.98	1.62
2018.03	0.17	35694.10	5745.89	25.38	19.00	647.06	-224.14	8.83	1607.47	1.66
2018.04	0.21	35408.50	5674.80	21.73	18.59	617.31	-250.65	8.64	1434.15	1.67
2018.05	0.24	34735.80	5759.02	19.27	18.40	609.62	-243.20	8.96	1349.57	1.63
2018.06	0.23	34318.90	5843.24	19.22	18.42	609.44	-243.93	9.55	1312.93	1.60
2018.07	0.25	34044.40	5927.46	19.15	18.56	609.62	-123.82	9.82	1241.13	1.58
2018.08	0.33	33942.30	5843.08	19.93	19.01	616.28	120.02	10.17	1216.51	1.57
2018.09	0.45	34213.80	5758.70	19.37	19.51	626.72	192.41	10.10	1125.15	1.58
2018.10	0.48	34248.00	5674.33	19.10	19.70	628.14	249.31	10.11	1091.34	1.61
2018.11	0.62	34008.30	5634.39	18.75	20.04	649.80	198.61	9.47	1086.60	1.64
2018.12	0.70	32750.00	5594.45	18.18	20.31	648.09	187.52	9.47	1068.23	1.65
2019.01	0.74	30883.20	5554.51	17.00	20.42	630.99	81.26	9.00	1087.88	1.64
2019.02	0.62	29215.50	5416.97	18.55	20.39	632.70	45.47	9.05	1107.25	1.62
2019.03	0.59	28864.90	5279.43	26.27	20.05	625.86	172.61	11.59	1199.46	1.60
2019.04	0.68	28027.90	5141.90	36.69	20.08	633.56	377.23	12.56	1465.42	1.58
2019.05	0.61	26850.70	4626.34	40.36	16.14	669.47	362.64	12.92	1722.04	1.59
2019.06	0.55	25481.30	4110.79	41.49	16.19	670.32	456.82	13.94	1742.70	1.60
2019.07	0.23	23086.10	3595.24	43.33	20.98	660.06	631.57	14.19	2202.58	1.60
2019.08	0.28	20823.60	3683.29	49.38	20.29	663.48	892.54	16.53	3203.15	1.54
2019.09	0.16	20198.90	3771.34	55.72	20.08	663.48	1073.75	19.45	3728.25	1.54
2019.10	0.18	20077.70	3859.39	77.69	20.03	763.42	2224.72	27.23	4993.03	1.55
2019.11	0.13	20479.30	3863.59	87.10	20.05	778.05	2163.88	24.82	6085.03	1.54
2019.12	0.10	20530.40	3867.79	81.93	20.06	779.05	2089.98	23.79	5207.24	1.53
2020.01	0.08	20427.80	3871.99	79.71	20.68	776.45	2025.57	26.42	5001.99	1.57
2020.02	0.10	20999.80	4199.47	87.86	22.50	790.02	2016.72	26.91	5148.43	1.57
2020.03	0.12	22208.50	4526.95	119.35	23.31	782.04	1362.60	28.16	5968.42	1.58
2020.04	0.12	23417.20	4854.42	133.36	23.53	809.17	1224.50	27.43	6095.72	1.59
2020.05	0.13	24330.40	4631.93	125.82	23.19	847.88	854.49	24.82	5301.67	1.59
2020.06	0.17	25230.70	4409.44	106.29	23.88	859.85	825.44	27.21	5350.38	1.59
